# Nanocomposite-Enabled Next-Generation Food Packaging: A Comprehensive Review on Advanced Preparation Methods, Functional Properties, Preservation Applications, and Safety Considerations

**DOI:** 10.3390/foods14213688

**Published:** 2025-10-29

**Authors:** Bo Peng, Xiaohui Qi, Linxiang Qiao, Jingting Lu, Ziyan Qian, Caie Wu, Zhaohui Xue, Xiaohong Kou

**Affiliations:** 1 School of Synthetic Biology and Biomanufacturing, Tianjin University, Tianjin 300072, China; pbo_1895@tju.edu.cn (B.P.); qxhqxx@tju.edu.cn (X.Q.); 2020214004@tju.edu.cn (L.Q.); ljt_777@tju.edu.cn (J.L.); qian_ziyan0117@tju.edu.cn (Z.Q.); 2 College of Light Industry and Food Engineering, Nanjing Forestry University, Nanjing 210037, China; wucaie@njfu.edu.cn; 3 State Key Laboratory of Synthetic Biology, Tianjin University, Tianjin 300072, China

**Keywords:** nanocomposites, food preservation, antimicrobial activity, barrier properties, safety assessment, chitosan, essential oils

## Abstract

This review comprehensively examines nanocomposite packaging materials for food preservation, focusing on their preparation methods, functional properties, applications, and safety considerations. Nanocomposites, incorporating nanomaterials such as metal nanoparticles, polysaccharides, or essential oils into polymer matrices, demonstrate enhanced mechanical strength, barrier properties (e.g., reduced water vapor and oxygen permeability), and significant antimicrobial activity. These advancements address critical food spoilage challenges by extending shelf life and maintaining quality in diverse products like fruits, vegetables, meats, and dairy. In addition, this review highlights concerns regarding potential cytotoxicity and migration of nanoparticles, underscoring the need for rigorous safety evaluations. While current methods (e.g., ionic gelation, electrospinning) show promise, scalability remains limited. Future research should prioritize eco-friendly designs, functional integration, and standardized safety protocols to facilitate commercial adoption.

## 1. Introduction

Global food security faces a critical challenge in the wake of substantial postharvest losses and widespread food waste. Current estimates suggest that roughly one-third (around 1.3 billion tons) of the total food produced annually for human consumption is wasted or lost within the supply chain [1]. This wastage not only represents a significant economic burden but also contributes substantially to environmental degradation, as decomposing organic matter in landfills generates methane emissions that exacerbate climate change [2,3]. The economic implications of this inefficiency are profound, with losses amounting to tens of millions of tons discarded annually by households, distributors, and retailers. In response to this pressing issue, the development of advanced food packaging technologies has emerged as a crucial strategy for mitigating food spoilage and extending the shelf life of perishable goods.

The limitations of conventional packaging materials, particularly petroleum-based polymers, have become increasingly apparent. These materials face growing scrutiny due to their non-biodegradable nature and contribution to environmental pollution [4]. While energy-intensive preservation approaches like cold-chain storage offer some protection, their high cost and operational complexity often limit accessibility, particularly in developing regions [5]. This technological gap has created an urgent need for more accessible and cost-effective strategies that can simultaneously address food preservation and environmental sustainability.

Recent advancements in nanotechnology have substantially broadened the scope of innovation within food packaging research. The integration of nanostructured materials has enabled the development of multifunctional packaging systems that extend beyond conventional preservation roles. Nanocomposite materials—engineered through the incorporation of diverse functional nanofillers—have emerged as promising alternatives to traditional packaging substrates, offering enhanced functional attributes and tunable properties [6]. These materials exhibit synergistic improvements in barrier capabilities, mechanical strength, and antimicrobial efficacy via the strategic incorporation of nanoscale constituents such as metallic nanoparticles, bioactive compounds, and hybrid organic-inorganic frameworks [7]. For instance, innovative approaches utilizing natural kaolin clay and Ficus carica-mediated silver nanoparticles (AgNPs) within chitosan matrices have demonstrated significant enhancements in mechanical and barrier performance [8]. Beyond metallic nanoparticles, research has expanded to include a wide variety of nanostructures including nanoemulsions of plant-derived essential oils, which exhibit pronounced antimicrobial activity against common foodborne pathogens [9].

The application spectrum of nanocomposite coatings and films has widened considerably, showing remarkable efficacy in shelf-life extension for diverse food products. Recent developments include quercetin-based nanocomposites that demonstrate exceptional antimicrobial performance (inhibition rates > 90% against *E. coli* and *S. aureus*) coupled with significant antioxidant activity (DPPH clearance rate of 80.7%) [10]. Similarly, advanced edible coating systems incorporating alginate–cinnamon essential oil composites have achieved shelf-life extension of up to 7 days for sliced chicken products while effectively inhibiting pathogenic microorganisms including E. coli and Listeria monocytogenes [11]. These innovations highlight the expanding potential of nanostructured materials to address multifaceted challenges in food preservation while simultaneously addressing environmental sustainability concerns.

Despite these significant advancements, several critical challenges impede the widespread commercialization of nanocomposite packaging technologies. Scalability remains a predominant constraint, as many synthesis methods lack the robustness required for industrial-scale production. Regulatory frameworks concerning nanoparticle migration and human exposure require further refinement, particularly regarding long-term safety assessment and standardized testing protocols [12,13,14]. Consumer acceptance also presents a substantial hurdle, with perceptions regarding nanotechnology in food contact applications varying significantly across demographic segments. Additionally, comprehensive toxicological profiling of nanomaterials necessitates further investigation, with particular emphasis on cytocompatibility, migration dynamics, and environmental impact assessment.

This comprehensive review aims to systematically evaluate the current landscape of nanocomposite materials for food preservation (NCFPs), examining their classification schemes, synthesis methodologies, functional properties, preservation mechanisms, and safety profiles. We critically analyze recent scientific advancements with particular emphasis on structure–function correlations and antimicrobial mechanisms. Furthermore, we discuss prevailing challenges and future prospects for NCFPs, providing insights that may guide the development of next-generation intelligent and sustainable packaging systems. Through this systematic examination, we aim to contribute to ongoing efforts toward reducing global food waste while promoting environmentally responsible packaging solutions.

## 2. Classification and Synthesis Strategies of NCFPs

### 2.1. Classification and Constituents: Matrices and Functional Fillers

Nanocomposite food packaging materials are structured around a matrix and functional fillers, with at least one component present at the nanoscale. When nanomaterials—such as nanochitosan or nanostarch—are employed as the matrix, the resulting composites often exhibit enhanced functional attributes due to their high specific surface area and nanoscale effects. Such nanomatrix composites are commonly fabricated through techniques such as mechanical grinding, high-pressure homogenization, or ionic gelation [15]. For instance, chitosan-based nanocomposites produced via ionic gelation demonstrate superior antibacterial and barrier properties compared to their conventional counterparts [16,17]. These improvements highlight the potential performance benefits of employing nanoscale matrices over microscale or molecular-scale alternatives.

Despite these advantages, the practical application of nanomatrix composites in food preservation remains constrained by challenges in scalable synthesis and processing. Currently, only a limited range of biopolymers—primarily certain polysaccharides and proteins—can be feasibly converted into nanomatrix forms, as illustrated in Table 1. Consequently, a more prevalent and technically viable strategy involves dispersing nanoscale materials as functional fillers within conventional polymer matrices.

Based on matrix composition, nanocomposites can be categorized into protein-based [55], polysaccharide-based [56], synthetic polymer-based [57], and compound matrix systems [58]. Synthetic polymers, in particular, encompass a diverse array of chemical structures and are often distinguished from biobased matrices in both sourcing and properties. Similarly, fillers can be classified into several types, including clay [26], organic matter [33], bioactive substances [59], metals (or their oxides) [60], and carbon nanostructures [46], as summarized in Table 1. To further enhance material performance, recent studies have explored the incorporation of multiple functional fillers to exploit synergistic effects. This multi-filler strategy can improve key properties such as mechanical strength, barrier performance, and antimicrobial activity [50]. For instance, one study demonstrated the integration of chitosan, oleic acid, TiO_2_, neem powder, and silver nanoparticles in hydroxypropyl methylcellulose (HPMC) films, with comparative analysis revealing distinct contributions from each filler type toward overall performance enhancement [61].

### 2.2. Preparation Methods

Nanoparticle synthesis strategies are broadly categorized into top-down (physical) and bottom-up (chemical or biological) approaches. In top-down processes, bulk materials are mechanically reduced to nanoscale dimensions. For instance, mechanical grinding using specialized equipment such as a Masuko grinder has been employed to produce nano-cellulose from bagasse pulp through repeated processing cycles [62]. Similarly, high-pressure homogenization and ultrasonication are widely utilized for their efficiency in nanoparticle formation with minimal side reactions, applicable to both individual nanoparticles and more complex nanocomposite systems [5,9,63,64].

In contrast, bottom-up methods, particularly redox-based chemical reactions, constitute another prevalent route for nanoparticle fabrication. A typical synthesis involves the hydrothermal treatment of precursors, as demonstrated in the preparation of ZnO nanoparticles from zinc sulfate and gallic acid under controlled temperature and pressure [65]. Likewise, graphene oxide (GO) is commonly synthesized via oxidation of graphite using potassium permanganate [46]. To mitigate the use of harsh chemical oxidants, eco-friendly biological synthesis pathways have been developed. For example, extracellular biosynthesis using fungal components (e.g., *Fusarium oxysporum*) has been successfully applied to produce silver nanoparticles (bio-AgNPs) incorporated into biodegradable polymer films [66]. Comparative studies between chemical and biological synthesis routes have indicated that biologically synthesized nanoparticles, though occasionally larger in size, can exhibit superior antibacterial efficacy [45].

Notably, the preparation of nanocomposites poses greater challenges compared to the synthesis of nanoparticles alone, primarily due to the complexities associated with achieving uniform dispersion and satisfactory interfacial compatibility. The key methods employed and their corresponding characteristics are summarized in Table 2.

#### 2.2.1. Ionic Gelation

**Figure 1:**
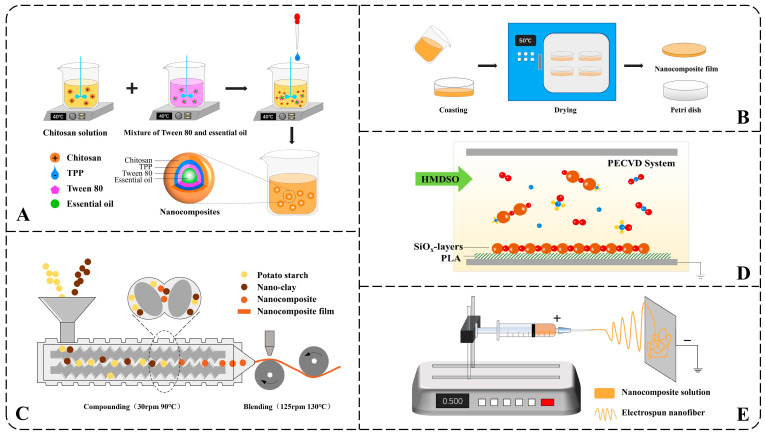
Schematic illustration of common preparation methods of nanocomposites: (**A**) ionic gelation method, (**B**) tape casting method, (**C**) injection-molded, (**D**) PECVD, (**E**) electrospinning method.

The ionic gelation method spontaneously forms nanoparticles through positive and negative charge attractions under stirring. Nanocomposites based on chitosan were often prepared in this way [59,71]. As can be seen in Figure 1A, chitosan with positive charges dissolved at a certain temperature. A filler, such as essential oil mixed with the emulsifier, was added to the chitosan solution. Subsequently, sodium tripolyphosphate (TPP) was dropped into the well-mixed solution under stirring. With stirring, chitosan (+) collides with TPP (−) and forms nanocomposites. Agitation speed, solution concentration, pH, and temperature all affect the size and properties of nanocomposites [67]. Therefore, nanocomposites can be prepared as needed by adjusting the parameters above without using large-scale equipment. However, the liquid nanocomposites and discontinuousness limited the method of scaling up to the industrial scale.

#### 2.2.2. Tape Casting

Tape casting is a method for forming dry membranes and is usually used after ionic gelation. The nanocomposite solution was cast into a container and dried (Figure 1B). Tape casting is a simple but often time and energy-consuming process.

Multilayer composite method can be applied as tape casting when repeated at least twice and requires drying time and energy consumption. It can also be used to develop better barrier properties. But as time went on, the multilayer composite films tend to delaminate, causing problems such as pinholes, cracks, surface non-uniformity, and reduced cohesion [5].

#### 2.2.3. Injection-Molded

Injection-molded method heats and melts the raw materials and then compacts them together through mechanical extrusion to prepare nanocomposites [38]. Compared to Ionic gelation and Tape casting, Injection-molding is easier to achieve mass production, but the requirements for processing and drying equipment are also higher (Figure 1C).

#### 2.2.4. Vapor Deposition

Vapor deposition is a traditional film preparation method that has been widely used in the semiconductor industry [72,73]. According to the nature of the process, gas-phase deposition can be classified into two major categories: chemical vapor deposition (CVD) and physical vapor deposition (PVD). Due to the advantages of the low substrate, fast deposition rate, and good film-forming properties, the method is also used in packaging. Research indicates that an environmentally friendly composite film can be prepared using the Plasma-Enhanced Chemical Vapor Deposition (PECVD) process. In this process, the reaction monomer hexamethyldisiloxane (HMDSO) is oxidized by oxygen under PECVD conditions, resulting in a uniform deposition on the polylactic acid (PLA) substrate (as seen in Figure 1D). The formed SiOx layer has a thickness of about 100 nm, effectively preventing the migration and diffusion of diethylene glycol dibenzoate (DEDB) into food simulants [69].

#### 2.2.5. Electrospinning

Electrospinning and electrocoating, technologies based on electrohydrodynamic principles, have gained prominence as effective methods for nanomaterial fabrication. They had been reported in various fields such as heat insulation [74], filtration [75], electromagnetic interference shielding [76], drug delivery [77] and desorption [78] but relatively rare in food preservation. Studies have demonstrated the potential of electrospun nanomaterials in enhancing food shelf life. For instance, ferulic acid-loaded zein/polyethylene nanofibers produced via electrospinning have been shown to significantly suppress decay in apples [79]. First, precursor solutions containing polymers, solvents, and catalysts must be prepared, whose stability directly affects the properties of electrospinning. Then, the water in the solution is evaporated using electrospinning under high voltage, and the nanofibers are formed [80] (Figure 1E). Similarly, environmentally friendly packaging materials have been developed using electrospun nanofibers (NFs) of black chickpea protein isolate (BCPI) incorporated with citral-loaded nanoliposomes (NLPs) [81]. In another approach, polycaprolactone/chitosan (PCL/CS) nanofiber membranes containing 10% Chinese yam polysaccharide (CYP) were fabricated, effectively delaying dehydration and spoilage in tomatoes [82].

#### 2.2.6. Coaxial Electrospraying

In contrast to conventional electrohydrodynamic approaches, coaxial electrohydrodynamic technology enables the simultaneous processing of two distinct polymer solutions into core–shell nanostructures that encapsulate active compounds. This method significantly enhances nanoparticle encapsulation efficiency and stability. This technique has been effectively utilized to synthesize antioxidant-loaded nanoparticles, such as betaine-gelatin systems, which exhibit high functional performance [83]. Furthermore, gelatin nanoparticles encapsulating *Stenocereus thurberi* extract have been fabricated via coaxial electrospinning and incorporated into sodium alginate-based coatings. These functional films demonstrate significant potential for preserving lipid-rich foods, including meat products, through controlled release of antioxidants [34].

In addition to the mainstream methods mentioned above, ultrasonic and infrared methods can also be used to prepare nanocomposites but usually play an auxiliary role [84]. Nonetheless, most electrohydrodynamic approaches remain largely confined to laboratory-scale development, indicating a substantial need for scaling efforts and industrial translation.

## 3. Structural and Performance Characterization of Nanocomposites

Microstructural and physicochemical characterization is essential for understanding the structure–property relationships of nanocomposites. Morphology and internal structure are two fundamental aspects that significantly influence material performance. Key morphological features—including particle size distribution, surface topography, and porosity—directly affect critical properties such as mechanical strength and barrier functionality. Empirical studies have confirmed, for example, a discernible correlation between nanoparticle size and elongation at break in various nanocomposite systems [85]. Thus, comprehensive microstructural analysis provides vital insights for the rational design of high-performance nanocomposites.

### 3.1. Micromorphological Analysis

**Figure 2:**
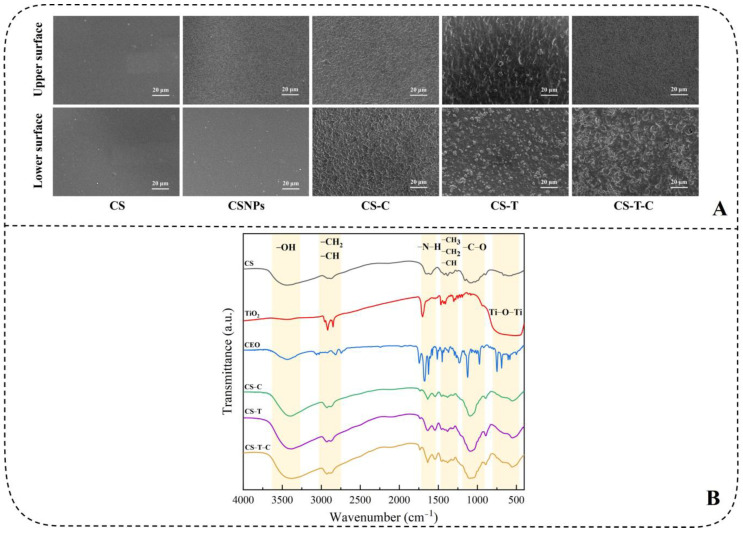
(**A**) SEM images of different nanocomposites. Upper surface: contacting with air; Bottom surface: contacting with the petri dish and (**B**) FTIR spectra patterns of pure CS, modified TiO_2_, CEO, CS-C, and CS-T-C [86].

Scanning electron microscopy (SEM) analysis reveals that the surface morphology of nanocomposites significantly influences their functional performance. As demonstrated in Figure 2A [86], the fabricated composite films retained structural continuity but developed protrusions under high filler loading, a morphological shift consistent with reported behavior under similar processing conditions [87]. This study attributes the observed asymmetry—specifically the enhanced roughness on the lower surfaces of modified films—to drying-induced molecular reorganization and nanoparticle aggregation. Unlike conventional mechanisms where pore formation results from essential oil migration and evaporation [85], the chitosan-based encapsulation utilized here effectively retained cinnamon essential oil (CEO), thereby altering microstructure development. Variations in component density further contributed to the final film topography [60,88], as evidenced by the granular texture of the CS-T film. Notably, the incorporation of CEO promoted a more uniform dispersion of TiO_2_ and improved surface homogeneity [18]. These structural characteristics support the application of the nanocomposites as functional edible coatings, in which a continuous upper layer serves as a barrier against moisture transfer while surface-localized active components enhance antimicrobial efficacy, thereby contributing to shelf-life extension.

### 3.2. Analysis of Microstructure and Binding Mechanisms

Fourier Transform Infrared Spectroscopy (FT-IR) is typically used to determine the functional groups and bonding modes of composite materials. This helps distinguish whether the bonding between different compounds depends on chemical bonding or physical adsorption. Currently, most nanocomposites are synthesized through hydrogen bonds and hydroxyl groups [55,56,89]. There was a change in the peak location and area before and after composites, but the overall infrared spectrum has no significant change [51]. As shown in Figure 2B [86], FT-IR analysis confirmed the successful chemical integration of CS, TiO_2_, and CEO into nanocomposites through specific bonding interactions. The intensified absorption at 3400 cm^−1^ in the CS-T-C spectrum suggests synergistic interactions between TiO_2_ and CEO. Characteristic peaks corresponding to Ti-OH bending at 1700 cm^−1^, C=O stretching from CEO aldehydes and phenols around 1674–1620 cm^−1^, and C-O-C vibrations between 1090–1036 cm^−1^ further verify chemical bonding [87,90]. Notably, the absence of significant change in the Ti-O-Ti vibration at 670 cm^−1^ implies preserved TiO_2_ integrity. These findings collectively demonstrate that the components are bonded chemically rather than through physical adsorption, endowing the nanocomposites with stable and functional properties suitable for food preservation applications.

However, there were also some nanocomposites whose matrix and filling material connect only by physical adsorption, and the infrared spectrum does not change. A representative study investigated the cross-linking between PVA and aldehydes, specifically glyoxal or glutaraldehyde. This study showed a decrease in the hydroxyl stretching band (3100–3500 cm^−1^), which corresponds to the stretching of the -OH group in PVA. The reduction in peak area observed in the composites may be attributed to the formation of a hemiacetal structure. This occurs when the aldehyde group in the cross-linking agent reacts with a hydroxyl group in the PVA polymer chain. Meanwhile, due to the cross-linking of glyoxal or glutaraldehyde, the CO-peak was weakened at 1249 cm^−1^. However, the spectra of the nanocomposites were the same before and after the addition of AuNPs and GO. There were no new peaks generated or changed. Therefore, it can be inferred that the binding between PVA and glyoxal/glutaraldehyde depends on the chemical bond, while nanoparticles mainly depend on physical adsorption [46]. In summary, the binding mode of each component in the nanocomposites varied with the substance type and preparation method.

## 4. Functional Properties of Nanocomposites for Food Packaging

Properties are the premise of material applications and the index to evaluate the quality of materials. Fillers can have a positive impact on the properties of nanocomposites.

### 4.1. Mechanical Strength and Flexibility

The mechanical properties of food packaging are essential for its storage. Packaging with good mechanical properties is easy to extend and process. This can help avoid mechanical damage to food when it is transported and sold. Additionally, good mechanical properties can maintain the integrity and barrier of packaging materials, avoiding direct contact between food and the external environment.

The two main indices of the mechanical properties of NCFPs are tensile strength (TS) and elongation at break (EB). TS refers to the maximum stress of material fracture under unidirectional uniform tensile load. EB refers to the ratio of the elongated part of the sample at the time of breaking to the initial length, which indicates the toughness of the film. As far as the film is concerned, the higher the TS and EB, the better the mechanical properties.

Incorporation of nanoparticles frequently enhances these mechanical properties. For example, the addition of gallic acid-functionalized ZnO nanoparticles into chitosan matrices has been shown to increase TS and EB by factors of seven and five, respectively, attributable to strong interfacial interactions such as hydrogen bonding [65]. Similarly, composites with TiO_2_, ZnO, or Ag nanoparticles demonstrate a 30–50% increase in tensile strength [35]. This may be caused by strong interactions between the matrix and nanoparticles, such as hydrogen bonding. However, the mechanical strength of nanocomposites does not always increase with the increase in nanoparticle concentration. It begins to decline when the concentration exceeds a certain range. Excessive nanoparticle loading—such as TiO_2_ beyond 1.5% in whey protein isolate/cellulose nanofiber composites—can induce aggregation, interfacial slippage, and network disruption, leading to a decline in mechanical properties [87,91]. In addition, the shape of the nanoparticles and the kinds of plasticizers or surfactants also affect the mechanical strength. Chitosan with Ti/ZnO nanorods/SiOx had better TS than chitosan with Ti/ZnO nanoballs/SiOx, but the opposite was true for EB [60]. This may be due to nanorod interweaving to form a network structure, whereas nanoballs fill the surface defects of chitosan. As for surfactants, sodium dodecylbenzene sulfonate performs better than sodium hexametaphosphate in improving TS and EB of nanocomposites [60]. However, the TS and EB of nanocomposites may exhibit an opposite trend with an increase in fillers [92].

### 4.2. Water Vapor Permeability (WVP)

Water vapor permeability (WVP) refers to the ability of water vapor to pass through a material under certain conditions. It is mainly influenced by diffusivity and water vapor solubility. The first depends on the porosity and tortuosity of the material, and the other depends on the hydrophilia [93]. For fresh food, packaging with a low WVP can maintain the water content and reduce weight loss. For dried food, it can prevent dampness.

Studies using single-factor experimental designs have revealed that WVP is highly sensitive to matrix composition and filler integration. For instance, low concentrations of chitosan (<2.0%) in whey protein blends result in insufficient cross-linking and elevated WVP, whereas high concentrations (>2.5%) introduce excess hydrophilic groups that increase water absorption and vapor transmission [50]. The addition of nanoparticles can increase the path of water vapor penetration and reduce the WVP through the denser structure composited with the matrix or by introducing hydrophobicity. However, agglomeration at high filler concentrations can disrupt matrix homogeneity and facilitate vapor permeation [91]. Nanofilaments similarly enhance barrier performance by extending diffusion pathways [33]. Unlike TiO_2_ and nanofilament, essential oil reduced WVP mainly through its hydrophobicity. While *rosemary* essential oil effectively limits water vapor transfer, excessive concentrations can lead to structural defects and increased WVP, even surpassing that of the unfilled polymer [26,87].

### 4.3. Oxygen Permeability

Oxygen permeability (OP) critically influences food quality: low oxygen levels retard lipid oxidation in fatty foods (such as walnuts) but may induce anaerobic spoilage in fruits and vegetables. Nanocomposites reduce OP by creating a zigzag path that hinders gas diffusion [27,42]. Gas transport is also affected by molecular interactions. For example, oxygen exhibits higher permeability than carbon dioxide in some nanocomposites due to its greater hydrogen-bonding capacity, facilitating its dissolution-diffusion through the polymer matrix [60]. Relatively few studies exist on OP in NCFPs, and this subject needs to be further studied and explored. Table 3 lists the physical properties of NCFPs.

### 4.4. Antimicrobial Efficacy

#### 4.4.1. Antibacterial Effects

Microorganisms are regarded as one of the main factors leading to food spoilage. Some microorganisms produce poisonous substances and harm human health [96]. The excellent antimicrobial properties of nanocomposites distinguish them from ordinary packaging materials. Several studies on developing nanocomposites with antimicrobial properties have been conducted [55,63,97]. Encapsulation of antimicrobial agents—such as peppermint and green tea oils—into nanoemulsions or nanocarriers improves their stability, prolongs activity, and enhances penetration into microbial cells [15]. Studies involving chitosan functionalized with Au or Ag nanoparticles have demonstrated broad-spectrum inhibition against Gram-positive (e.g., *S. aureus*) and Gram-negative bacteria (e.g., *Pseudomonas aeruginosa*), fungi (e.g., *Aspergillus niger*), and yeast (e.g., *Candida albicans*) [98]. Antifungal applications have also proven effective; nanochitosan with cinnamon oil delayed fungal growth in cucumbers until day 9 of storage, compared to day 4 in untreated controls. Therefore, the addition of antimicrobial fillers can enhance the breadth and strength of the antimicrobial properties of NCFPs.

#### 4.4.2. Antimicrobial Mechanisms

**Figure 3:**
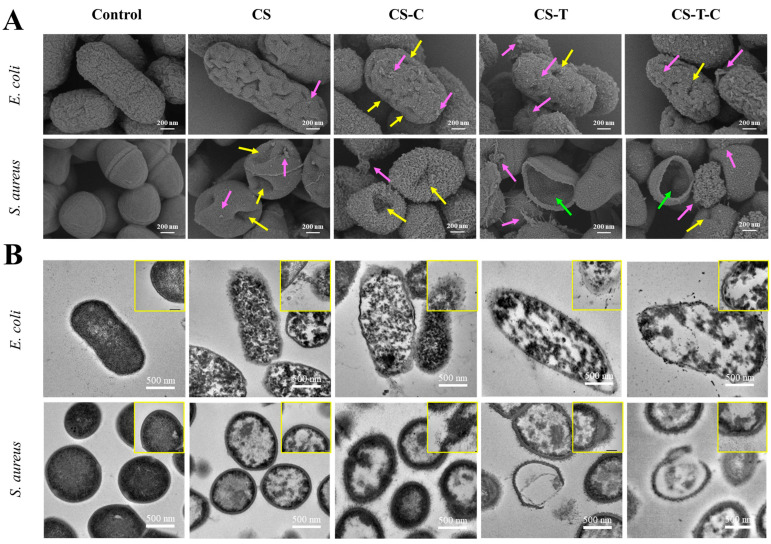
Analysis of the morphology of bacteria treated with different nanocomposites: (**A**) the SEM and (**B**) the TEM morphologies of *E. coli* and *S. aureus* [86].

The antimicrobial mechanisms of nanocomposites are multifaceted and often synergistic. As shown in Figure 3, the *E. coli* and *S. aureus* treated with nanocomposites showed tiny bits on the surface in common, accompanied by holes. Some cell contents even flowed out from the weak part of the cell wall. The cell wall was broken, and the contents completely flowed out, leaving only empty shells. This study showed that the prominent surface effect of nanocomposites may have more collisions and reactions with the cell wall surface, resulting in multiple damages to cells and accelerated death [86].

**Figure 4:**
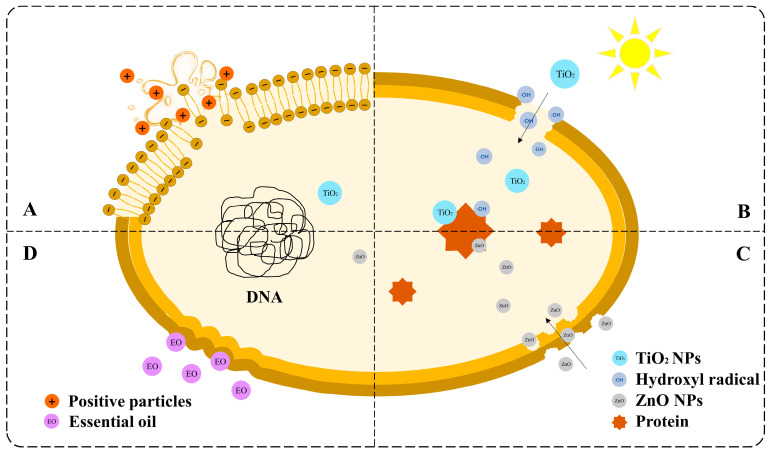
Schematic diagram of the antibacterial mechanism of nanocomposites: (**A**) the charge attraction theory, (**B**) free radical injury, (**C**) direct contact and membrane disruption, and (**D**) essential oil-mediated mechanisms.

The antibacterial mechanism of nanocomposites is controversial. The four primary mechanisms are shown in Figure 4.

The first common mechanism is the charge attraction theory (Figure 4A). Bacteria with negative surface charges and nanocomposites with positive charges, such as chitosan and metal ions, can be attracted together. The structure of the cell membrane is then destroyed, causing the contents to leak and the microorganisms to die [99]. The second most common mechanism was free radical injury (Figure 4B). TiO_2_ exhibits photocatalytic activity, which can be activated using ultraviolet (UV) radiation. Pairs of electrons and holes are separated in the conduction and valence bands, respectively [100]. Subsequently, water and oxygen generate free radicals with strong oxidation, such as ·OH, which strike the cell membrane and cause damage and death. ZnO nanoparticles are also believed to exert bacteriostatic effects. However, another mechanism is that ZnO nanoparticles can directly interact with the cell wall and enter the cell interior (Figure 4C). ZnO nanoparticles then bind to proteins and other biomacromolecules, interfering with the normal physiological activities of cells [96,101]. The fourth mechanism is natural antimicrobial substances (Figure 4D). Essential oils containing esters and terpenes are typically added to nanocomposites to enhance their antimicrobial activity. Lipophilic esters and terpenes can cause damage to the structure of microorganism cell walls, decreasing cell respiration rate [102,103].

There are also many other explanations for the antimicrobial mechanisms of the nanocomposite. For instance, the insoluble chitosan molecules form a waterproof chitosan coating on the cell surface, preventing the transmission of nutrients and causing cell death [99]. Multiple antimicrobial mechanisms may coexist in a single system because of the diversity of the nanocomposites and microbial species. The specific mechanism of action requires further study. This will also become the basis for the design of NCFPs in the future.

### 4.5. Antioxidant Efficacy

In addition to antimicrobial protection, controlling oxidative spoilage is critical for maintaining the quality and shelf life of lipid-rich and pigment-sensitive foods. The integration of antioxidant-active nanofillers provides a robust strategy to mitigate oxidative deterioration induced by reactive oxygen species (ROS) [104].

#### 4.5.1. Antimicrobial Mechanisms

The primary antioxidant mechanisms of nanocomposites include free radical scavenging, metal ion chelation, and UV barrier effects.

##### Free Radical Scavenging

Many natural compounds incorporated as nanofillers, such as curcumin, quercetin, and essential oil constituents (e.g., thymol and carvacrol), possess phenolic structures that donate hydrogen atoms to neutralize peroxyl radicals (ROO·) and other free radicals, thereby interrupting lipid oxidation chain reactions [40,104].

##### Metal Ion Chelation

The antioxidant functionality of certain biopolymer-based nanocomposites is partly attributed to their capacity to chelate pro-oxidant metal ions such as Fe^2+^ and Cu^2+^. Chitosan, for instance, exhibits metal-binding properties that suppress the catalytic activity of these ions in Fenton-like reactions, thereby reducing the generation of highly reactive hydroxyl radicals (·OH) [99].

In multi-cross-linked chitosan films incorporating caffeic acid and Fe^3+^, the antioxidant effect is primarily mediated through the formation of stable coordination complexes between Fe^3+^ and the catechol groups of caffeic acid. This chelation mechanism effectively sequesters free Fe^3+^ ions, thereby inhibiting their participation in Fenton and Haber–Weiss reactions—key pathways for ·OH generation from hydrogen peroxide and superoxide. By suppressing metal-ion-catalyzed reactions, the film impedes the initiation and propagation of radical-mediated lipid peroxidation. Furthermore, the integration of Fe^3+^-caffeic acid complexes within the cross-linked network enhances structural cohesion and immobilizes the chelating agents, ensuring sustained antioxidant efficacy without significant leaching. This strategic incorporation of specific chelating motifs within a robust polymer matrix offers a viable approach for designing advanced antioxidant packaging systems [105].

##### UV Barrier

Nanofillers like TiO_2_, ZnO, and carbon nanostructures effectively block UV light, a primary initiator of photo-oxidation. By reducing UV penetration, these nanocomposites protect photosensitive food components from radiolytic degradation [35,46].

#### 4.5.2. Antioxidant Effects

The effectiveness of antioxidant nanocomposites has been validated across various food matrices. For example, active films containing quercetin exhibited a DPPH radical clearance rate of 80.7%, effectively delaying oxidative browning in fruits [10]. In another study, ferulic acid-loaded zein/polyethylene oxide nanofibers applied to apples significantly suppressed superficial scald and decay, attributes linked to their antioxidant capacity [79]. These findings underscore the potential of nanocomposites to function as integrated preservation systems, addressing both microbial and oxidative spoilage pathways simultaneously.

A significant advantage of NCFPs lies in the synergistic efficacy of multi-functional nanofillers. For instance, essential oils such as cinnamon and clove oil not only disrupt microbial cell membranes but also contain high levels of phenolic compounds that act as potent antioxidants [15,17]. This dual functionality was demonstrated in a chitosan-based nanocomposite incorporated with clove essential oil, which simultaneously inhibited microbial growth and significantly reduced lipid oxidation in pomegranate arils during storage [17]. Similarly, gelatin nanoparticles encapsulating *Stenocereus thurberi* extract within a sodium alginate coating provided concurrent antioxidant and antimicrobial protection for meat products [34].

## 5. Application in Food Preservation: From Fresh Produce to Animal-Derived Products

Polystyrene (PS), polypropylene (PP), polyvinylchloride (PVC), polyethylene terephthalate (PET), and other petroleum-based plastics are widely used in food packaging. However, these materials do not retain freshness of the food. Biologically based nanocomposites with good mechanical and barrier properties and antimicrobial activity are regarded as alternatives to petroleum-based plastics, not only in their properties but also in a variety of applications and performances.

### 5.1. Application of Nanocomposites in Common Foods

Many studies have reported that nanocomposites can effectively maintain the quality of fruit [105], vegetable [106], meat [107], fish [108], dairy product [109], etc. Table 4 lists the applications of NCFPs on different kinds of food.

Fruits are a popular food variety to be preserved using nanocomposites. Preservation of whole fruit with skin [117], berries [39], fresh-cut salad [118], or pulp [119], whether climacteric fruit [120] or non-climacteric fruit [121], have been reported. These fruits could be stored 2–3 times longer using the nanocomposites. However, few studies have been conducted on fruits with a long shelf life, such as apples. Compared to fruits, fewer studies have focused on vegetable preservation using nanocomposites. For instance, a composite coating consisting of konjac glucomannan, carrageenan, and nano-SiO_2_ was shown to effectively maintain the whiteness, appearance, and hardness of white mushrooms over 12 days [111]. Similarly, nanofibrous films composed of polylactic acid (PLA), TiO_2_, and graphene oxide (GO) delayed the red ripening and softening of green peppers by at least 6 days [90]. Nevertheless, preservation technologies for leafy vegetables remain underexplored, primarily attributable to their intrinsic characteristics such as short growth cycles, limited storage requirements, and high susceptibility to physical damage during coating application. These challenges are further compounded by high water activity, which adversely affects the adhesion and integrity of coatings, as well as potential migration risks and stability issues in high-moisture environments [44,90].

The main reason for meat preservation is inhibition of microorganisms. NCFP with outstanding antimicrobial activity has been applied to almost all common meat categories, such as chicken [122], pork [48], beef [123], and fish [124]. Similarly, dairy products are susceptible to microbial contamination. Dairy products such as cheese have been coated with sodium alginate-based nanocomposites incorporated with mandarin fiber and oregano essential oil, significantly improving microbial stability and nutritional retention [114].

For dry fruits, the barrier property of the packaging is an important indicator. Comparative studies have indicated that nanocomposite films based on banana flour and garlic essential oil can extend the shelf life of roasted peanuts under high-temperature conditions (45 °C) to 11 weeks, better preserving product quality and serving as an effective alternative to traditional PET packaging [22].

The application of nanocomposites in the preservation of liquid food such as milk is rarely reported [125,126]. Improving the stability of nanocomposites for long-term contact with liquids may be key to their development.

### 5.2. Different Forms of Nanocomposite Materials in Food Applications

The performances of the nanocomposites also vary. Coating is the most widely used method for NCFP. The process is simple and material-saving; the food only requires soaking in the nanocomposite solution for a short time, followed by drying. Nanocomposites for food packaging are implemented in various forms, each offering distinct functional advantages. Coatings are the most widely used form due to their simplicity and material efficiency; foods are typically dipped into nanocomposite solutions and dried. Alternatively, nanocomposite films can be sold as a standalone product and are also convenient for consumers to use, which helps enhance product appeal and value. Active packaging papers functionalized with nanocomponents also constitute an emerging category of functional materials [100]. In addition, there is also a type of NCFP that does not come into direct contact with food [23]. The nanocomposites were packaged in separate bags away from the fruits. The fruits were kept fresh by the release of bacteriostatic nanoparticles. This may be more acceptable for consumers who doubt the safety of nanocomposites. However, the optimal performance of these nanocomposites has not yet been reported. This could be a direction of nanocomposites for future research.

## 6. Safety Evaluation of Nanocomposites in Food Packaging

The safety of food packaging materials is of paramount importance, particularly with the incorporation of nanomaterials. While significant research has focused on the functional properties of nanocomposites, studies specifically addressing their safety remain relatively limited. Current safety assessments primarily rely on migration tests or biotoxicity evaluations [19,82,86,127].

### 6.1. Migration Testing

Migration of nanoparticles from packaging into food constitutes a critical safety concern. The process typically occurs in two phases: initial release of surface particles upon direct contact, followed by gradual diffusion of embedded particles from the matrix into the food substance [4]. This migration behavior generally adheres to Fickian diffusion principles, influenced by factors including nanocomposite density, crystallinity, cross-linking degree, food composition, and environmental conditions [4,128]. Experimental studies have revealed a biphasic release profile characterized by an initial burst release followed by a sustained slow release phase. The incorporation of certain clay minerals such as laponite has been shown to effectively reduce silver nanoparticle migration [88]. Comparative analysis using food simulants and real food matrices showed that the mobility of nanomaterials was significantly reduced in complex food systems. For example, the migration levels of titanium and silver nanoparticles in the food mimics were 112.92 and 135.58 μg/kg, respectively, while they were only 14.59 and 20.04 μg/kg in the cheese matrix. Notably, all reported values remain below the European Food Safety Authority (EFSA) threshold of 10 mg/kg [44]. The controlled release of active substances represents an essential functionality of certain antimicrobial nanocomposites, some of which primarily rely on the release of such agents during storage to achieve preservative effects such as antimicrobial or antioxidant activity (such as essential oils) [129], thereby extending food shelf life. Consequently, precise regulation of release kinetics and quantitative analysis of migration levels are critical considerations in the design of nanocomposite materials. Furthermore, as the stability of some active compounds can be influenced by external environmental factors (such as anthocyanins) [130], potential transformations of nanoparticles during migration warrant careful investigation.

### 6.2. Biotoxicity Assessment

Biotoxicity testing provides direct evidence regarding the safety of nanocomposites. In vitro cytotoxicity assessments using mouse pancreatic cancer cells (Panc02) demonstrated cell viability exceeding 90% after 24-hour exposure to nanocomposite concentrations ranging from 0.1 to 1 μg/mL [27]. In vivo evaluations employing roundworms have yielded additional safety insights. Survival rates of roundworms elegans fed with nanocomposite-coated bell pepper juice showed no significant difference compared to those consuming fresh bell pepper extract [45]. However, contradictory findings indicate that certain clay components in nanocomposites may exert toxic effects on Caco-2 and HepG2 cells, with toxicity profiles dependent on clay type, concentration, and specific cell line characteristics [131].

### 6.3. Regulatory Frameworks and Safety Assessment

Nanocomposites are generally prepared using legally permissible materials and additives; however, the properties of nanosized materials are difficult to assess. The large specific surface area and biological activity may give nanocomposites new and undesirable properties [4]. For example, AgNPs can cross the blood–brain and placental barriers and exhibit significant toxicity in neuronal precursor cells at concentrations of 5 mg/L and lower [132]. In addition, it is controversial whether some nanocomposites that achieve antimicrobial effects by releasing active substances belong to the classification of food packages or additives.

The regulatory landscape for nanomaterial applications in food contact materials continues to evolve [4]. The EFSA Scientific Committee published a set of guidelines in August 2021, named the Guidance on Risk Assessment of Nanomaterials to be Applied in the Food and Feed Chain: Human and Animal Health. Compared to previous versions, this set of guidelines provides insights into the physicochemical properties, exposure assessment, hazard characteristics, and applications of nanomaterials. Exposure assessment, hazard identification, and characterization have been introduced and discussed in detail [12,133]. The US did not regulate the definition of nanomaterials, nanotechnology, or other related terms until 2018 [13]. At present, China has not approved nanofood or raw materials and has not formulated relevant standards and regulations. Thus, it is necessary to develop laws and regulations that cover the definitions, properties, preparation methods, detection methods, and safe doses of nanocomposites.

## 7. Conclusions and Future Perspectives

In summary, this review emphasizes that nanocomposites for food packaging (NCFPs) primarily rely on polysaccharide- and protein-based matrices—such as chitosan, starch, gelatin, and whey protein—due to their biocompatibility, biodegradability, and excellent film-forming properties [4,92]. These renewable materials align with the growing demand for sustainable packaging alternatives to conventional plastics [60]. However, the transition from lab-scale production to industrial manufacturing remains a major obstacle, hindered by issues such as nanoparticle aggregation, thermal instability, and high operational costs [5]. Scaling up requires optimized and energy-efficient processes such as electrospinning, extrusion, and solvent casting, with better control over reproducibility and economic feasibility [65].

NCFPs exhibit significantly enhanced functional performance, including improved mechanical strength, superior gas and moisture barrier properties, and notable antimicrobial activity [35,48,87]. Despite these advances, fundamental structure–property relationships demand further investigation—particularly nanofiller dispersion, matrix–filler interfacial compatibility, and durability under practical conditions. Moreover, industrial adoption necessitates improving critical properties such as heat sealability and printability, potentially through surface modification or hybrid nanofiller strategies [45].

Although NCFPs have been effectively applied to prolong the shelf life of fruits, meat, and dairy products, their use remains limited for cereals, leafy vegetables, and liquid foods [111]. These challenges stem from specific product characteristics: high water activity in leafy vegetables affects coating adhesion and integrity, while liquid foods pose migration risks and stability concerns. Future designs should account for application-specific requirements, such as controlled release mechanisms for dry foods and enhanced hydrophobicity or composite layering for moist environments.

Regulatory frameworks for nanomaterials in food contact applications are still under development. Current guidelines from agencies like EFSA and FDA lack comprehensive protocols for assessing nanoparticle migration, chronic exposure, and long-term toxicological impacts [12,133]. There is an urgent need for international harmonization of testing standards and legal definitions to ensure safety and regulatory compliance [13]. Future research should focus on migration mechanisms, degradation pathways, and potential health effects using robust in vitro and in vivo models.

In conclusion, while NCFPs represent a promising frontier in smart and sustainable food packaging, several challenges—including scalable production, material compatibility for diverse foods, and regulatory uncertainty—must be addressed. Interdisciplinary collaboration across material science, toxicology, and food engineering is essential to advance these innovations. Looking forward, intelligent packaging systems with real-time freshness monitoring and biosensing capabilities offer exciting pathways to reduce food waste and enhance safety throughout the supply chain.

## Figures and Tables

**Table 1:** Different types of NCFPs.

Type of Film/Coating	Matrix	Filler	Ref.
**Nanomaterials as matri** **x**			
Protein	Nano-whey protein concentrate	Corn oil and TiO_2_	[18]
	Gelatin nanofibers	Sesamol	[19]
Polysaccharide	Nanochitosan	CaCl_2_	[20]
	Nanochitosan	Green tea oil Peppermint oil	[15]
	Bacterial nanocellulose	Cinnamaldehyde	[21]
Polysaccharide	Banana flour starch nanoparticles	MMT and garlic essential oil	[22]
Others	Nanomontmorillonite	Ag	[23]
Compound matrix	Carboxymethylcellulose (CMC), nano-cellulose fiber (NCF), and nanochitosan (N-Ch)	Green pomelo peel essential oil (GPO)	[24]
**Nanomaterials as filler**			
Protein	Gelatin	Nanocerium oxide	[25]
	Nile tilapia (Oreochromis niloticus) protein isolate	Oregano essential oil and nano-clay clove essential oil and nano-clay	[26]
	Egg white and egg yolk albumen protein	Cellulose nanocrystal and curcumin	[27]
	Whey protein	Cellulose nanofiber and *rosemary* essential oil and TiO_2_	[28]
Polysaccharide	Alginate	Lysozyme	[29]
	Carrageenan	Nano-ZnO	[30]
	Chitosan	peanut shell nano-lignocellulose (NLC)	[31]
	Chitosan	Cinnamaldehyde-tannic acid zinc acetate nanoparticles	[32]
	K/ι-Hybrid Carrageenan	Cellulose nanowhiskers	[33]
	Sodium alginate	Gelatin nanoparticles encapsulating pitaya extract (Stenocereus thurberi)	[34]
	Starch	Nano-TiO_2_	[35]
Polysaccharide	Starch	Chitin nanocrystal Chitin nanofiber	[36]
	Loquat seed starch	Resveratrol core–shell biopolymer nanoparticles	[37]
	Potato starch	Clay	[38]
	Pullulan	Cinnamon essential oil	[39]
	Pectin	Curcumin and essential oil	[40]
Synthetic polymer	Biaxially oriented polypropylene (BOPP)	ZnO nanorods Spherical ZnO	[41]
	High-density polyethylene	Marigold flower extract and TiO_2_	[42]
	Polyethylene terephthalate (PET)	Cellulose nanocrystals	[43]
	Polylactic acid (PLA)	TiO_2_ and Ag	[44]
	Polyvinylpyrrolidone (PLV)	AgNPs	[45]
	Polyvinyl alcohol (PVA)	AuNPs Graphene oxide	[46]
Others	Shellac	TiO_2_	[47]
Compound matrix	Chitosan and gelatin	Tarragon essential oils	[48]
	Soy protein isolate (SPI) and fenugreek seed gum (FSG)	*P. graveolens* extract	[49]
	Chitosan and whey protein	Nano-cellulose and cinnamaldehyde	[50]
Compound matrix	K-carrageenan and hydroxypropyl methylcellulose	Nisin and nano-rhamnose	[51]
	PVA and CMC	TiO_2_	[52]
	PLA, sodium alginate, and chitosan	Al_2_O_3_	[53]
	Chitosan and polyvinyl alcohol	AgNPs and purple sweet potato anthocyanins	[54]

**Table 2:** Characteristics of common nanocomposite fabrication methods.

Fabrication Method	Advantages	Disadvantages	Exemplary System	Ref.
Ionic gelation method	Simple, mild reaction conditions, without large-scale equipment.	Liquid, long reaction time, difficult to industrialize.	Chitosan and thymol	[67]
Tape casting	Simple, without large-scale equipment.	Long time, high-energy consumption, and discontinuous.	Chitosan, nano-ZnO, and gallic acid	[65]
Multilayer composite	Simple, without large-scale equipment.	Long time, easy to delaminate.	PVA, sodium alginate, and chitosan	[68]
Injection-molded	Simple, quantity production.	Need specialized equipment, unsuitable for heat-sensitive materials.	Potato starch and nano-clay	[38]
Chemical or physical vapor deposition	Mature technology, low substrate, fast deposition rate, uniform density, and good film-forming properties.	Complex chemical reactions, need specialized equipment, mostly used for metals and their oxides.	PLA and nano-SiO_2_	[69]
Electrospinning method	Excellent performance, new technology, widely applicable.	High voltage, need specialized equipment.	Zein nanofibers and curcumin	[70]

**Table 3:** Physical properties of NCFPs.

Composition	Mechanical Property		Barrier Property		Ref.
	TS (MPa)	EAB (%)	WVP (mm g/h m^2^ kPa)	OP (cm^3^ mm/h m^2^ kPa)	
**Protein**					
Egg protein isolate	2.5–15.0	3.0–16.5	~1.125	~31.66	[27]
Egg protein, cellulose nanocrystal, and curcumin	3.3–3.5	10–14	~0.417	~8.75	
Gelatin	~36.5	~26.8	0.4316 ± 0.0220	NA	[91]
Gelatin and TiO_2_	~42.5	~49.6	0.3596 ± 0.01112	NA	
Whey protein concentrate	0.66 ± 0.09	55 ± 9	0.622 ± 0.051	NA	[18]
Nano-whey protein concentrate	0.82 ± 0.05	84 ± 8	0.571 ± 0.095	NA	
Whey protein concentrate and TiO_2_	0.55 ± 0.06	58 ± 3	0.663 ± 0.103	NA	
Nano-whey protein concentrate and TiO_2_	0.70 ± 0.03	73 ± 3	0.611 ± 0.119	NA	
Whey protein isolate (WPI)	13.07 ± 0.03	76.61 ± 0.07	0.105 ± 0.000	NA	[87]
WPI and cellulose nanofiber (CNF)	15.85 ± 0.05	62.14 ± 0.05	0.087 ± 0.000	NA	
WPI and CNF and TiO_2_	18.61 ± 0.02	59.04 ± 0.03	0.078 ± 0.000	NA	
WPI, CNF, TiO_2_, and *rosemary* essential oil	17.03 ± 0.01	62.08 ± 0.02	0.045 ± 0.001	NA	
**Polysaccharide**					
Carrageenan	84.83 ± 4.67	60.94 ± 6.03	2.745 ± 0.064 (g/m^2^ day)	NA	[30]
Carrageenan and ZnO NPs (0.5%)	121.53 ± 6.57	65.48 ± 1.49	2.556 ± 0.038	NA	[30]
Carrageenan and ZnO NPs (1%)	113.07 ± 4.66	65.91 ± 2.49	2.498 ± 0.036	NA	
Chitosan (CS)	0.1133 ± 0.0093	11.07 ± 0.3889	9.113 ± 0.1411	9.487 ± 0.2304	[65]
CS and ZnO@gal (30 mg)	10.47 ± 0.2899	13.19 ± 0.2192	3.065 ± 0.0586	8.772 ± 0.2091	
CS and ZnO@gal (70 mg)	54.83 ± 0.1414	52.17 ± 0.2192	1.176 ± 0.2157	5.570 ± 0.3051	
Chitosan (CS)	50.61 ± 0.45	4.32 ± 0.01	3.542 ± 0.125 (kg/m^2^ d)	1.2917 ± 0.0017 (cm^2^/d kPa)	[94]
CS and Ti/ZnO nanorods/SiOx	84.21 ± 0.16	4.60 ± 0.04	3.000 ± 0.250	0.9583 ± 0.0042	
CS and Ti/ZnO nanoballs/SiOx	72.86 ± 0.62	7.30 ± 0.08	3.042 ± 0.125	1.1667 ± 0.0004	
Pullulan	53.3 ± 3.9	3.0 ± 0.5	78 ± 6	NA	[84]
Pullulan and cinnamon oil	49.3 ± 0.6	5.4 ± 0.4	50 ± 4	NA	
Starch	5.74 ± 0.22	35.84 ± 2.39	~0.623	NA	[35]
Starch and TiO_2_ (1%)	5.51 ± 0.47	40.44 ± 3.28	~0.497	NA	
Starch and TiO_2_ (3%)	5.34 ± 0.71	42.50 ± 4.05	~0.367	NA	
Starch and TiO_2_ (5%)	5.27 ± 0.49	50.94 ± 3.56	~0.407	NA	
**Synthetic polymer**					
PBAT	8.78 ± 0.49	347.77 ± 4.95	~0.117	~5.20	[95]
PBAT, MgO, and AgNPs (1%)	11.91 ± 0.18	415.63 ± 2.87	~0.047	~3.80	
PBAT, MgO, and AgNPs (5%)	6.27 ± 0.32	187.22 ± 16.12	~0.036	~3.46	
PVA	0.47 ± 0.41	118.42 ± 5.86	54.24 ± 2.67 (g/m^2^ h)	NA	[46]
PVA and AuNPs	1.45 ± 0.07	34.79 ± 1.56	33.11 ± 1.65	NA	
PVA and graphene oxide	1.51 ± 0.07	96.97 ± 4.81	32.13 ± 1.73	NA	
**Compound matrix**					
Chitosan	27.28 ± 1.39	20.59 ± 0.93	0.0860 ± 0.0022	0.28 ± 0.02	[42]
Chitosan and TPGS	25.54 ± 1.50	32.22 ± 1.65	0.0752 ± 0.0011	0.11 ± 0.01	
Chitosan, TPGS, and SiO_2_	32.99 ± 0.91	40.53 ± 0.97	0.0626 ± 0.0036	0.09 ± 0.01	
PVA	23.20	291.23	0.1516	NA	[52]
PVA and CMC	25.21	215.36	0.1292	NA	
PVA, CMC, and TiO_2_	33.50	270.54	0.1746	NA	

**Table 4:** Applications of NCFPs.

Application	Composition	Performance	Effect	Ref.
**Fruit**				
Chinese cherry	Chitosan and nano-SiOx	Coating	Reduce weight loss by 51%, decay rate by 32% and increase firmness by 57%.	[110]
Pomegranate arils	Chitosan NPs and clove essential oil	Coating	Maintain the sensory and nutritional qualities of pomegranate, extend the shelf life by 54 days.	[17]
Mango	Carrageenan and ZnO	Coating	Avoid bacterial Black Spot in 33 days of storage.	[30]
Grape Plum	Chitosan, HPMC, TiO_2_, neem-doped chitosan, HPMC, and Ag NPs	Film	Extend the shelf life of the grape by 10 days and the plums by 3 weeks.	[61]
Banana	PVA and glyoxal and AuNPs	Film	With the formation of minimum black spots in 5 days.	[46]
Kiwifruit and pineapple fresh salad	Montmorillonite and Ag	Nanoparticles	Extend the shelf life by more than 5 days.	[23]
**Vegetable**				
Cucumber	Chitosan and *Cinnamomum zeylanicum* essential oil	Coating	Firmer, maintain color, water content, lower microbial counts, and extend the shelf life up to 21 d at 10 ± 1 °C.	[102]
Fresh-cut pepper	PVP and AgNPs	Coating	Extend the shelf life to 12 days at 4 °C without any other damage.	[45]
White mushrooms	Konjac glucomannan, carrageenan, and nano-SiO_2_	Coating	Maintain the whiteness, visual appearance and hardness and extend the shelf life by 5 to 12 days.	[111]
Green pepper	PLA, TiO_2_, and GO nano-fibrous	Film	Delay the green pepper to turn red and soften and inhibit microbial corruption.	[90]
Button mushroom	Nanochitosan and *Citrus aurantium* essential oil	Nanoparticles	Increase the activity of GR and APX and decrease the microbial count of button mushrooms during the 15-day storage period.	[112]
**Meat, Fish, and Seafood**				
Silver carp fish balls	Chitosan, ZnO, TiO_2_, and SiOx	Coating	Not corrupted until the 24th day.	[60]
Chicken filets	Pectin, curcumin, and curcumin–cinnamon essential oil	Coating	Delay the appearance of microbial spoilage and extend the shelf life to 12 days.	[40]
Lamb meat	Whey protein isolate, cellulose nanofiber, TiO_2_ nanoparticle, and *rosemary* essential oil	Film	Extend the shelf life by 9 days.	[28]
Chicken breasts	LDPE, ZnO, and Ag	Film	A lower depletion of oxygen and lower microbial counts than control packaging.	[113]
**Dry fruit**				
Chestnut	Chitosan, whey protein, nano-cellulose, and cinnamaldehyde	Coating	Reduce the weight loss rate, mildew rate, and calcification index.	[50]
Roasted peanuts	Banana flour and garlic essential oil	Film	A good alternative to PET.	[22]
**Others**				
Cheese	Sodium alginate, mandarin fiber, and oregano essential oil	Coating	Inhibit the growth of psychrophilic bacteria, molds, and yeasts and prolong shelf life.	[114]
Egg	PVA, sodium alginate, and chitosan	Coating	Maintain A grade for 15 days.	[68]
*G. biloba* seeds	Chitosan and nano-SiO_2_ Chitosan and nano-TiO_2_	Coating	Inhibit mildew occurrence, shrinkage, and maintain the firmness, positively affecting the antioxidant activity.	[89]
Steamed buns	Mung bean starch, soluble soybean polysaccharide, and cinnamon and clove essential oil	Coating	Extend the shelf life of steamed buns from the normal 3 days to 10 days, at 10 °C.	[115]
Apple juice	PLA, Ag NPs, and Vitamin E	Film	The absorbance, conductivity, and pH do not vary obviously after 48 h, while other samples change soon.	[116]
Clarified butter	Cellulose, chitosan, and Ag/TiO_2_	Active paper	Extend the shelf life to 9.3 months.	[100]

## Data Availability

Data is contained within this article.
